# Merkel Cell Carcinoma: Interdisciplinary Management of a Rare Disease

**DOI:** 10.1155/2013/189342

**Published:** 2013-01-14

**Authors:** Sven Schneider, Dietmar Thurnher, Boban M. Erovic

**Affiliations:** Department of Otolaryngology—Head and Neck Surgery, Medical University of Vienna, Währinger Gürtel 18-20, 1090 Vienna, Austria

## Abstract

*Background*. The goal of this paper is to review contemporary multidisciplinary treatment with reference to Merkel cell carcinoma. Management of this rare but highly aggressive skin cancer is a complex undertaking that necessitates an understanding of its etiology, epidemiology, clinical presentation, and the coordinated work of several clinical specializations. *Recent Findings*. The contemporary literature employs a multidisciplinary approach to achieve the best patient's treatment. *Conclusion*. This paper presents an algorithm for contemporary management for the rare and aggressive Merkel cell carcinoma. Multidisciplinary approach in a tumor center provides high-quality care for patients with Merkel cell carcinoma.

## 1. Introduction

Merkel cell carcinoma (MCC) is a rare neuroendocrine skin tumor, with a high risk of local and distant spread. The incidence of MCC is 0.32 per 100.000 [1], showing an increasing incidence with advanced age and in male Caucasians [2]. 

Progression in incidence might be linked to the constantly increasing exposure to ultraviolet B radiation considering the fact that MCCs are localized frequently in sun-exposed areas of the body. Other known risk factors are immunosuppression in transplant recipients [3], HIV [4], and in particular Merkel cell carcinoma polyomavirus infection [5].

The head and neck area is the most frequently affected site (29–40.6%), followed by extremities (21–38%), trunk (7–23%), and unknown primary sites (3.4–12%) [6–8]. Unfortunately, clinical appearance of MCC is heterogeneous. It frequently presents as an asymptomatic, reddish, bluish, or purple tumor of the skin. Size at the time of first consultation is usually smaller than 2 cm, although MCC is characterized by rapid growth [9]. Due to the long list of, mostly, benign skin tumors, diagnosis based on clinical parameters is challenging. A recently performed study showed that in 56% of patients with MCC a benign tumor was initially presumed, mirroring the problems in clinical examination and challenges in clinical diagnosis [8]. However, diagnosis is finally achieved by histopathological analysis of small biopsies or samples of totally excised tumor.

Additionally, people's awareness of this disease is very low compared to malignant melanoma. This suggestion might be supported by the observation that most patients are seen with an advanced stage of disease. 

The 5-year survival rate ranges from 30 to 64% [6, 10], although survival is strongly dependent on the presence of regional and distant metastasis with a far worse outcome in advanced stages of disease. About 50% of patients showed localized stage of disease at the initial presentation. A recent single institution study shows the impact of stage of disease on 5-year survival showing MCC-specific survival of 87, 63, 42, and 0% for stages I, II, III, and IV, respectively [11]. 

High awareness to this rare cancer type among the population as well as among physicians can provide the key to early diagnosis. Besides the consideration of clinical risk factors, improved diagnostic tools like specific protein markers in immunohistochemistry [12] increased significantly the rate of diagnosis in Merkel cell carcinoma patients. Hence, improvement in diagnostics should be accompanied by optimization of multidisciplinary treatment strategies to deliver high-quality cancer care for patients with Merkel cell carcinoma. 

The rareness of MCC accompanied by the lack of outcome reports as well as relative treatment inconsistence raises further barriers to general treatment recommendations. However, there is evidence of improvement in recurrence and survival rate due to either adjuvant radiotherapy or chemotherapy following surgical management [13, 14]. Furthermore, in an early stage patients not receiving multimodality treatment increased locoregional recurrence was observed [15, 16]. Thus, multidisciplinary management of MCC appears as the most favorable approach. 

In this paper we will review the contemporary interdisciplinary management of patients with Merkel cell carcinoma and present our experience at the University of Otolaryngology, Head and Neck Surgery, Vienna.

## 2. Multidisciplinary Management

MCC is an extraordinary rare disease. Thus, there is still little knowledge to guide the care of patients with MCC. Furthermore, limited data on interdisciplinary treatment evaluation and outcome analysis of multidisciplinary decision-making exist in general.

Although until now there is no evaluation of tumor board decisions in patients with Merkel cell carcinoma, several other types of cancer multidisciplinary discussions on patients showed relevant impact on their clinical outcome. In ovarian cancer it could be shown that management by a multidisciplinary team at a joint clinic significantly increased patients' survival [17]. Also in gastroesophageal cancer, patients managed by a multidisciplinary team were more likely to survive 5 years compared to patients who were managed independently by surgeons [18]. Furthermore, several studies have shown that discussion in tumor board conferences altered the final diagnosis [19, 20], led to treatment alterations [21] or changes in management [22], and improved staging accuracy [23].

Treatment of MCC often requires a wide field of specialties like dermatologists, head and neck surgeons, radiooncologists, oncologists, pathologists, radiologists, speech pathologists, and nursing which goes along with extensive coordination management.

Although a heterogeneous field of therapeutic strategies exists, wide resection of the tumor followed by sentinel lymph node biopsy is standard treatment. According to pathological examination, total lymph node dissection is frequently performed. Surgical treatment is carried out by dermatologists and head and neck surgeons. Furthermore, oncologists and radiooncologists are frequently involved in adjuvant therapy in patients with advanced stage of disease. Thus, the key for successful management of patients with this highly aggressive disease is a multidisciplinary clinic, at which coordination of care with multiple medical specialties is established [24]. High-quality care for patients with MCC as well as their relatives can be provided in an interdisciplinary setting. 

According to the aims of the Institute of Medicine of the National Academics, high-quality care must follow six proposed aims [25]. Care must be safe, meaning that injuries by the treatment have to be free of avoidable errors. Care must be effective by providing services based on scientific knowledge to all who could benefit. Moreover, care is supposed to be patient centered within the meaning of respectfulness and response to individual patient preferences, needs, and values. A timely and efficient process characterizes high-quality cancer care. Waits and delays as well as waste of equipment, supplies, ideas, and energy must be avoided. Finally, providing care must be equitable in terms of consistence in quality and independence of sociodemographic characteristics. 

Considering the need for multidisciplinary evaluation, special attention should be paid to time-efficient clinical evaluation and patients' treatment according to high-quality cancer care aims. Coordination of care among specialists is considered as essential for high-quality oncologic care, whereas lack of coordination is a main drawback for patients' treatment and improvement of care [26]. 

One of the essential cornerstones in treatment of MCC is a multidisciplinary tumor board for implementation of the goals of high-quality treatment in an interdisciplinary fashion. 

Management of all cancer patients should be discussed and planned in multidisciplinary meetings, due to the fact that it facilitates ensuring quality of care and decreasing organizational difficulties in the treatment of cancer [27]. According to the French Cancer Plan, a definition for multidisciplinary meetings has been established [28], emphasizing main quality criteria. First, a multidisciplinary approach means that specialists from at least three medical disciplines have to be present. Formal structure concerning frequency of meetings, paperwork, and conclusion reports must be given. Moreover, it is essential that every cancer case must have a conclusion report, in which medical decisions must be based on clinical practice guidelines. Board recommendations must be communicated to the patient to implement therapeutically decisions. Importantly, recommendations of multidisciplinary meetings must be periodically evaluated. 

Another benefit of multidisciplinary management is cost efficiency. Although no cost analysis of a multidisciplinary setting of MCC patients is currently available, it has been demonstrated for melanoma treatment that multidisciplinary care at a large academic medical center can be more cost efficient than a less organized traditional community-based approach [29]. It leads to the assumption that cost reduction is also possible in MCC treatment by specialists in an academic, multidisciplinary setting. 

It is favorable that each patient is presented in this board as soon as possible after histologic diagnosis for further discussion of treatment options. During the last decades, cancer treatment shows an increasing complexity. Regarding the progressing specialization as well as more sophisticated treatment options in every discipline involved in cancer treatment, planning of high-quality therapeutic approaches is not possible for an independent physician. According to this way of thinking, multimodal treatment is a consequence of interdisciplinary discussion and planning. Clearly, development of therapeutic strategies in a tumor board is the most time efficient way to enhance patient management by gathering experts of each discipline, but also long-term effects on patients' outcome have to be considered. 

Despite the benefits of multidisciplinary management, there are also several pitfalls in this setting. Noteworthy, there is no standardized expert panel for several cancer types. This may influence therapeutic decisions by the presence or absence of a certain specialist and might reflect personal preferences. Furthermore, definition of being an expert of a certain specialty is rarely given. No standardized qualification criteria for attending a tumor-board as a decision maker so far exists.

Considering these facts, treatment decisions may depend on the presence or absence as well as on the qualification of several specialists, which can make it hard to relate to certain decisions. 

Particularly in Merkel cell carcinoma, it is important to arrange a setting in which treatment options can be discussed and recommendations are well documented. Due to the rarity of MCC, the lack of prospective clinical studies and conflicting literature on the treatment and outcome of Merkel cell carcinoma, standardized management is often not established. Taking one step forward, one can say that high-quality care and improvement of treatment are only provided in a multidisciplinary, academic setting. The number of patients to collect data in an effort to improve patient care as well as clinical and basic research might not be obtained outside a multidisciplinary center. 

Therefore, a multidisciplinary approach in a center, most favorable in an academic setting, is the only possibility to provide high-quality care as well as improvement of therapeutic strategies so patients with this rare and aggressive disease benefit the most.

## 3. Case Report

To illustrate the need for multidisciplinary management we consecutively describe the case of a 77-year-old women who was diagnosed with Merkel cell carcinoma in April of 2012. Her medical history included treatment for a melanoma on the leg in 1997 and CLL since 2007. 

Initially, this patient was seen in a private praxis by a dermatologist. She had a slow growing nodular tumor above her left eyebrow. Unfortunately, clinically this tumor was not suspicious for a malignancy and thus an open biopsy was carried out. As soon as the histological workup showed an R2 resection of a Merkel cell carcinoma, the patient was sent to the outpatient clinic at a department of dermatology in Vienna. At this time, the tumor measured 1.5 cm in diameter and was localized superior of the right eyebrow, paramedian, and close to the supratrochlear vessels. Subsequently, in May 2012 wide local resection of the tumor with sentinel lymph node biopsy was carried out. The primary site was closed with full thickness skin harvested from the right chest.

At the primary site, resection margins were negative, however, the sentinel node, localized in the ipsilateral parotid gland, was positive for Merkel cell carcinoma. Staging by computed tomography of the head and neck, thorax, and abdomen was conducted after the sentinel node biopsy. Imaging showed that the patient had at least two intraparotideal lymph node metastases and multiple ipsilateral cervical lymph nodes highly suspicious for metastatic disease.

**Figure 1:**
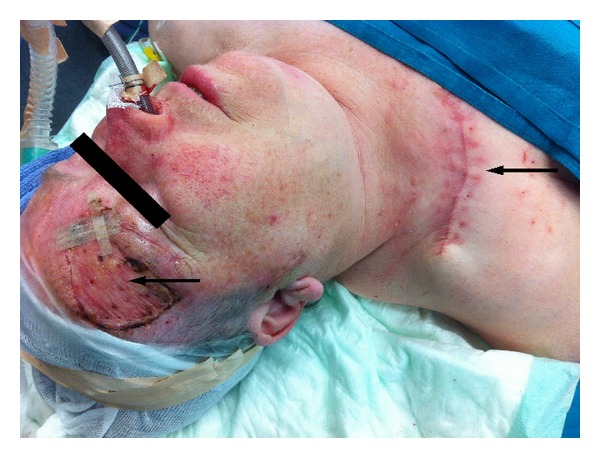
Patient with Merkel cell carcinoma of the right eyebrow (arrow). Primary excision site was covered by full thickness skin taken from the right clavicular/subclavicular region (arrow).

**Figure 2:**
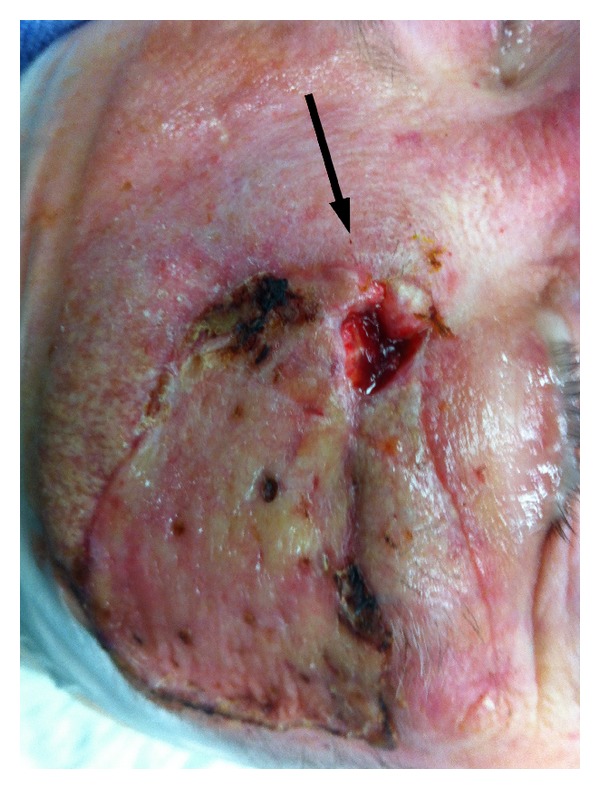
Wound dehiscence at the primary site (arrow).

**Figure 3:**
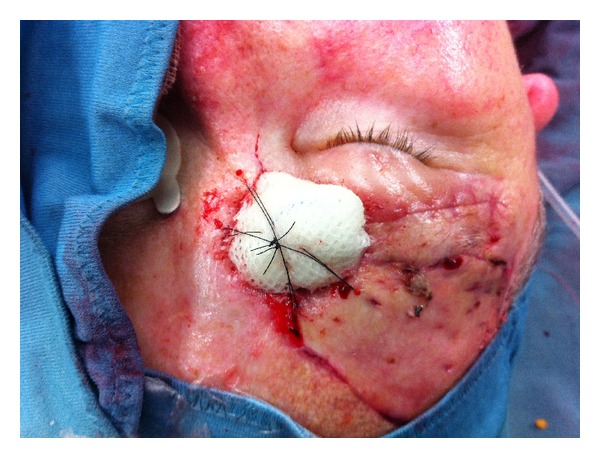
Wound dehiscence was biopsied and frozen sections showed to be positive for recurrent MCC disease. Carcinoma was resected again and STSG was used to cover the defect over the right eyebrow.

**Figure 4:**
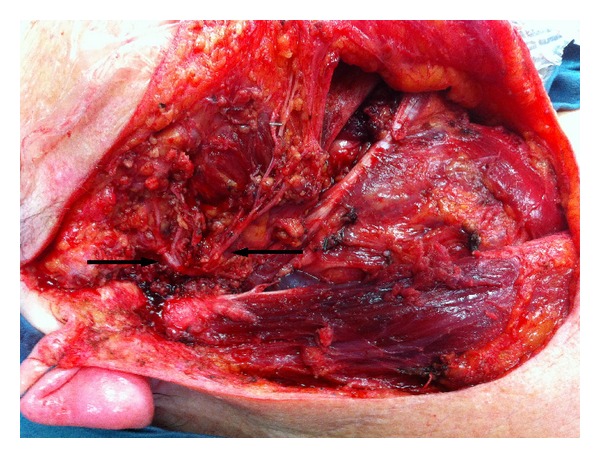
After right parotidectomy and selective neck dissection all branches of the facial nerve could be preserved (arrows).

**Figure 5:**
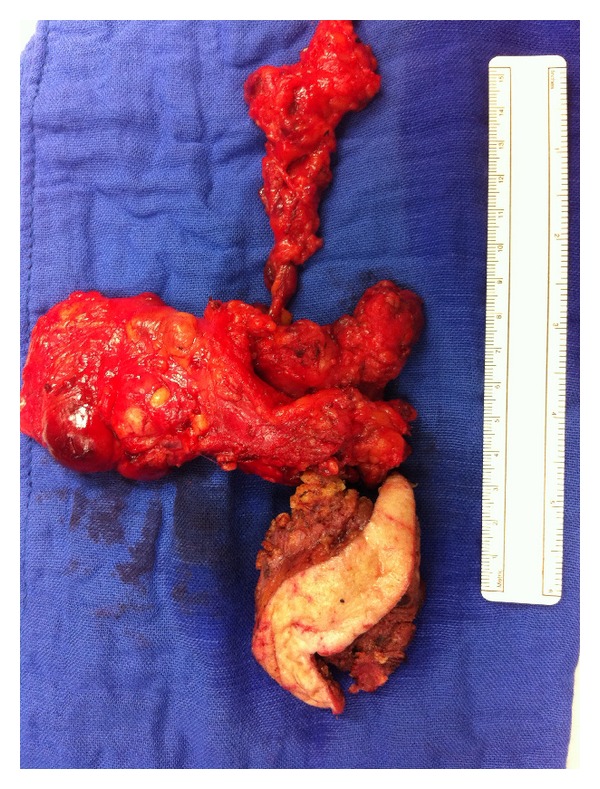
Parotidectomy and neck dissection specimen measuring 20 cm × 15 cm.

Two and a half weeks later the patient was seen at the Department of Otolaryngology, Head and Neck Surgery, Vienna Medical University, and was presented at the interdisciplinary tumor board for head and neck tumors. Therapeutic options were discussed as followed: either adjuvant radiotherapy at the primary site including the ipsilateral parotid gland and neck or surgery followed by postoperative radiotherapy. Meanwhile the patient developed a wound dehiscence at the primary tumor site, highly suspicious for local recurrence (Figures 1 and 2). The first line recommendation of the multidisciplinary board was to perform a subtotal parotidectomy, selective ipsilateral neck dissection followed by radiotherapy. The patient agreed and surgery was performed in June 2012. Intraoperatively biopsy from the wound dehiscence was carried out and pathological examination by frozen sectioning showed Merkel cell carcinoma. Again wide local resection was performed and the defect was closed with a split thickness skin graft (Figure 3). Subsequently, subtotal parotidectomy and selective neck dissection was performed (Figures 4 and 5). In the final pathology report the parotid gland was positive for Merkel cell carcinoma and multiple lymph nodes were infiltrated as well. In particular, level 1a showed 2 out of 13, level 1b 6 out of 6, level 2a 6 out of 8, level 2b 2 out of 3, level 3 6 out of 8, and level 4 16 out of 22 lymph nodes positive for Merkel cell carcinoma.

At the time of writing up the paper the patient finished adjuvant radiotherapy and is currently free of tumor disease.

## 4. What Could Have Been Done Better?

Although it is obvious that physicians always intent to provide high-quality treatment to their patients, the case report shows that there are several pitfalls in clinical work-up with Merkel cell carcinoma patients. According to the goals of high-quality treatment, effective, safe, equitable, and patient-centered treatment could be achieved, but this patients' medical history shows a lack of in time and efficient management.

In particular, at our institution such small tumors would be completely excised and sent to pathology. Imaging work-up is always initiated at the initial presentation of the patient and in particular before performing sentinel node biopsy. To the best of our knowledge, in this presented case we were not able not find any imaging that has been done before sentinel node biopsy. As a second open biopsy in the parotid gland was carried out that represents a significant drawback in regards to possible spread of tumor cell into surrounding tissue. 

In regards to waiting time, this case shows an unnecessary loss of time from the point of histological diagnosis to planning and initiating treatment. An early presentation of the patient in a multidisciplinary tumor board would have avoided loss of time as well as facilitated the planning of multidisciplinary treatment. 

## 5. Multidisciplinary Tumor Board for Head and Neck Cancer at the Medical University of Vienna

At the initial presentation of tumor patients a careful and meticulous examination of the head and neck, including endoscopic examination, is performed. Consecutively, an excision biopsy, depending on the size of the primary tumor, is taken under local anesthesia in the clinic. In case of suspicious lymph nodes, fine needle aspiration is performed. Additionally, all patients with skin malignancies are seen by a dermatologist. 

As a second step, an ultrasonography, CT or MRI of the neck, and, if possible, a PET-CT are carried out. With all histological and imaging reports patients are presented at the weekly tumor board for head and neck cancer. Patient's history and all diagnostic findings are presented either by a resident or attending physician. Presentation includes the medical history as well as the by the patient itself preferred therapy.

Best therapeutic strategy is discussed in a multidisciplinary approach among all members of the tumor board including head and neck surgeons, dermatologists, radiooncologists, oncologists, and radiologists. In case of the need of further examination or planning of therapy, appointments are made at the same meeting to provide time efficient management. 

Considering all the provided facts, the tumor board members give a treatment recommendation that will be offered to the patient and its family members at the next appointment. 

## 6. Conclusion

Management of Merkel cell carcinoma is a huge challenge for physicians and patients and their social surrounding.

In our case paper we could clearly show that the need for a multidisciplinary planning of therapy is highly time and cost efficient and linked to best-treatment outcome. Immediate presentation after histological diagnosis in a multidisciplinary setting can reduce waiting time for treatment. Furthermore, an interdisciplinary surgical approach can be planned and carried out and thereby reduce length of in-patient stays and frequency of surgery. 

For best patients' care, especially for patients with rare diseases, a multidisciplinary tumor board is the most favorable treatment tool.
